# Re: Postponing colonoscopy for 6 months in high‐risk population increases colorectal cancer detection in China

**DOI:** 10.1002/cam4.6227

**Published:** 2023-08-11

**Authors:** Ali Noursina, Farzad Safari

**Affiliations:** ^1^ School of Medicine Isfahan University of Medical Science Isfahan Iran

To the Editor, we are writing to respectfully raise a concern regarding the article titled “Postponing colonoscopy for 6 months in high‐risk population increases colorectal cancer detection in China,” which was recently published in your esteemed scientific journal.^1^

While we found the study to be informative and useful, we would like to draw your attention to an issue regarding the methodology that was employed in the research. Specifically, the article states that the patients who were examined were asymptomatic, yet their symptoms were assessed through a high‐risk factor questionnaire (HRFQ). This questionnaire included a history of chronic constipation, chronic diarrhea, and bloody mucous stools. However, it is not clearly stated in the paper how these symptoms were incorporated into the study or how they were assessed.

As a reader and a fellow researcher, we believe it is important to have a clear understanding of the methodology that was used in the study, particularly given its potential impact on this valuable work with such a large sample size. Therefore, we respectfully request that the authors provide more details on how the questionnaire was administered and how the symptom assessment was conducted.

Thank you for your attention to this matter. We appreciate your continued efforts to publish high‐quality scientific research.

## AUTHOR CONTRIBUTIONS

**Ali Noursina:** Conceptualization (equal). **Farzad Safari:** Conceptualization (equal); methodology (equal); writing – original draft (equal).

## Data Availability

Data sharing is not applicable to this article as no new data were created or analyzed in this study.
